# Redox Signaling and Its Impact on Skeletal and Vascular Responses to Spaceflight

**DOI:** 10.3390/ijms18102153

**Published:** 2017-10-16

**Authors:** Candice G. T. Tahimic, Ruth K. Globus

**Affiliations:** 1 Space Biosciences Division, NASA Ames Research Center, Moffett Field, CA 94035, USA; candiceginn.t.tahimic@nasa.gov; 2 KBRWyle, Moffett Field, CA 94035, USA

**Keywords:** spaceflight, bone, vasculature, oxidative stress, microgravity, hindlimb unloading, radiation, reactive oxygen species, antioxidant

## Abstract

Spaceflight entails exposure to numerous environmental challenges with the potential to contribute to both musculoskeletal and vascular dysfunction. The purpose of this review is to describe current understanding of microgravity and radiation impacts on the mammalian skeleton and associated vasculature at the level of the whole organism. Recent experiments from spaceflight and ground-based models have provided fresh insights into how these environmental stresses influence mechanisms that are related to redox signaling, oxidative stress, and tissue dysfunction. Emerging mechanistic knowledge on cellular defenses to radiation and other environmental stressors, including microgravity, are useful for both screening and developing interventions against spaceflight-induced deficits in bone and vascular function.

## 1. The Spaceflight Environment and Its Impact on Skeletal and Vascular Health

Microgravity and radiation are two unique elements of the spaceflight environment that pose challenges to the health of an organism. Microgravity leads to a cephalad fluid shift and profound reductions in mechanical loading of bone and muscle. Spaceflight causes perturbations in calcium homeostasis and site-specific reductions in bone mass (osteopenia), and thus may pose long-term risks for skeletal health and tissue repair [1,2,3,4]. In rodent models of weightlessness such as hindlimb unloading (HU), the onset of osteopenia correlates with reductions in skeletal perfusion, vascular density (rarefication), and vasodilation responses similar to that observed in aging [5,6,7]. These are serious risks for long-duration, exploration-class missions when astronauts will face the challenges of increased exposure to space radiation and abrupt transitions between different gravitational states upon return to Earth.

Beyond the Earth’s protective magnetosphere, astronauts will be exposed to a complex combination of ionizing radiation from galactic cosmic radiation (GCR) and intermittent solar particle events (SPEs). GCR is comprised of α particles, protons and a small percentage of high-charge and high-energy (HZE) nuclei while SPEs generate highly energetic protons and heavy ions. HZE particles are of particular concern during exploration-class missions [8] (reviewed in [9]). HZE easily penetrate spacecraft shielding and biological tissue, deposit very large amounts of energy along linear tracks (high linear energy transfer, high LET) that cause DNA strand breakage, and generate secondary radiations that may have additional detrimental biological effects [10,11,12,13,14]. Other responses to ionizing radiation include oxidative stress, damage to proteins, lipids, and DNA, adversely affecting functions of membranes, the extracellular matrix, cell cycle and survival [15,16,17].

## 2. Oxidative Stress and Its Link to Spaceflight-Induced Tissue Dysfunction

### 2.1. Oxidative Damage Associated with Spaceflight and Its Analogs

#### 2.1.1. Evidence from Spaceflight

Spaceflight and the return to Earth may lead to oxidative damage in blood and various tissues as a result of excessive reactive oxygen species/reactive nitrogen species (ROS/RNS) in both humans and animals [18,19,20,21,22,23]. Collectively, a number of studies indicate that various tissues undergo altered redox status during and/or after spaceflight. Urinary excretion of the oxidative damage markers, 8-iso-prostaglandin F2α (8-iso-PGF2α) and 8-hydroxydeoxyguanosine (8-OHdG) were measured in-flight (88 to 186 days in orbit) and post-flight (up to 14 days) in Mir mission crew [19]. The isoprostane, 8-iso-PGF2α, is a marker for oxidative damage to membrane lipids and is produced by the peroxidation of arachidonic acid in membrane phospholipids while 8-OHdG is an oxidized derivative of the nucleoside, deoxyguanosine, and is therefore used to assess oxidative damage to DNA. 8-OHdG excretion was unchanged during spaceflight and increased postflight. No changes in 8-OHdG levels were observed in Earth-based individuals that underwent bed rest although isoprostane was increased in the ensuing recovery period. Changes in isoprostane production were attributed to decreased generation of oxygen radicals from the electron transport chain due to reduced caloric intake in-flight, whereas the post-flight increases in the excretion of oxidative damage markers may be partly caused by a combination of increased metabolic activity following flight and the loss of some antioxidant defenses during flight. The downregulation of antioxidant defenses as a potential mechanism for the increased levels of oxidative damage post-flight is supported by the observation that hair follicle samples from International Space Station (ISS) crew members at post-flight display decreased expression of endogenous antioxidant genes, including Mn superoxide dismutase (MnSOD), CuZnSOD, glutathione peroxidase 4 (*GPX4*) and kelch-like ECH-associated protein 1 (*KEAP1*), the regulator of nuclear factor erythroid 2-related factor 2 (*NFE2L2* a.k.a. *NRF2*), a master transcription factor that regulates hundreds of oxidative defense-related genes [24].

Consistent with the findings in spaceflight crew members, rodents subjected to spaceflight also exhibit alterations in redox signaling relative to ground controls. In rats, short-duration spaceflight (6 days) increases cardiac gene expression of mitochondrial redox-related enzymes [25], suggesting a possible stress response and/or change in energy metabolism. Although short duration spaceflight (7 days) does not alter heart mass in rats [26], differences between flight and ground controls were observed in the vasculature of mice [27,28]. Spaceflight induces similar effects in the liver of both rats and mice, which includes elevated expression levels of antioxidant genes and markers of oxidative damage [18,29]. Male rats flown in STS-63 for eight days showed reduced total glutathione (GSH) content and activities of CuZnSOD (*SOD1*), catalase, GSH reductase, and GSH sulfur-transferase in liver [18]. In ocular tissue, astronauts are susceptible to optic disc edema, globe flattening, and choroidal fold formation [30]. The mechanisms are unknown, although mice flown in STS-135 also showed ocular tissue damage as well as alterations in several critical genes involved in regulating vascular endothelial cell response to oxidative stress and apoptosis [31].

#### 2.1.2. Evidence from Ground-Based Models for Spaceflight

Ground-based simulations of microgravity and space radiation also demonstrate a link between oxidative stress and tissue impairments. Rodents exposed to proton (50 cGy) or ^56^Fe (15 cGy) display increased oxidative damage in the heart and decrements in clinical measures of cardiac function [32] while γ-radiation exposure (^137^Cs, 1–2 Gy) increases ROS generation and lipid peroxidation in bone marrow, and decreases cancellous bone volume fraction [33]. Furthermore, bone marrow from rodents exposed to radiation display increased expression of inflammatory cytokines interleukin-6 (*IL6*), tumor necrosis factor alpha (*TNFα*), monocyte chemoattractant protein-1 (*MCP1*), and the pro-osteoclastogenic signal, receptor activator of nuclear factor kappa-B ligand (*RANKL*), as well as upregulation of *NRF2* [34,35].

Hindlimb unloading (HU) is a widely accepted rodent model to simulate weightlessness for many different tissues (reviewed in [36,37,38]). Marrow cells of the osteoblast lineage from HU mice show elevated ROS within a week of HU [39]. Marrow and bone show parallel increases in gene expression of the cytosolic fraction of the free radical scavenger, superoxide dismutase (*SOD*), changes that coincide with HU-induced reductions in osteoblast activity and bone loss [39]. HU also causes adaptation-related changes in cardiovascular function [40,41,42,43,44], and several studies have linked HU-induced vascular responses and oxidative stress [45,46,47]. Taken together, observations from both spaceflight and ground-based rodent models that simulate weightlessness show that oxidative stress and damage may occur in response to microgravity and radiation across multiple, if not all, tissues.

## 3. The Role of Nitric Oxide (NO) and Reactive Oxygen Species (ROS) Signaling in Skeletal and Vascular Disease

### 3.1. NO and ROS Signaling: Mechanisms and Impact on Tissue Function

Excess reactive oxygen species/reactive nitrogen species (ROS/RNS) and inflammation are implicated both in age-related diseases, such as osteoporosis and atherosclerosis, and following insults such as radiation exposure [15]. ROS can directly stimulate bone resorption by osteoclasts [48,49], and the bone loss due to aging and estrogen deficiency may be partly attributed to oxidative stress [50,51]. Furthermore, treatment with the potent antioxidant, α-lipoic acid, can prevent inflammation- and acute radiation-induced bone loss [33,52]. Oxidative damage from the production of excess ROS/RNS in skeletal tissues during spaceflight may lead to delayed deficits in bone structural integrity and reduced mechanical strength during recovery and the aging process.

Under physiological conditions, ROS and RNS such as nitric oxide (NO) can function as signaling molecules to regulate important processes such as mechanotransduction [53,54,55,56,57,58,59] and vascular function [60]. However, at high levels, ROS are potent inducers of apoptosis in response to a vast array of cellular insults [61,62]. Superoxide (O_2_^−^) in particular is cytotoxic and is produced when molecular oxygen reacts with an aqueous electron. O_2_^−^ can be generated by exposure of cells to ionizing irradiation and as a by-product of metabolism, primarily from mitochondria. O_2_^−^ is an upstream component of many oxidative pathways and can be rapidly converted into hydrogen peroxide (H_2_O_2_) by members of the superoxide dismutase (*SOD*) family of antioxidants [63,64]. Catalase, glutathione peroxidase (*GPX*), and other peroxidases such as peroxiredoxin then convert H_2_O_2_ into H_2_O [65]. H_2_O_2_ can react with endogenous Fe^2+^ or other transition metals via a fenton mechanism to generate the highly reactive hydroxyl radical (OH•) [66] which is one of the most damaging free radical species produced by exposure to ionizing radiation [67].

Normal vascular function requires both basal and stimulated production of NO in the endothelium by endothelial nitric oxide synthase (*eNOS*). The *eNOS* enzyme utilizes l-arginine to produce NO and l-citrulline. NO then activates soluble guanylate cyclase (*sGC*) by binding to it. This leads to increased production of cyclic guanosine monophosphate (cGMP) which in turn mediates vascular smooth muscle relaxation [68].

Genetic ablation studies of *eNOS* in rodents demonstrate the critical role of endothelial NO signaling in vascular health [69]. *eNOS* knockout (KO) mice lack endothelium-derived relaxing factor (*EDRF*) activity in response to endothelium-dependent vasodilators (e.g., acetylcholine) [70,71]. Furthermore, they are hypertensive [71], display increased vascular smooth muscle cell proliferation [72], and platelet aggregation [73], as well as a higher predisposition to atherosclerosis [74], thrombosis [75], and stroke [69,76,77].

The activity of *eNOS* is regulated via complex and concerted processes, including transcriptional control, substrate availability, interactions with other proteins and co-factors and post-translational modification [69]. Under conditions of oxidative stress, the biological activity of NO may decline in resistance arteries. For example, in the presence of O_2_^−^, NO rapidly combines to form peroxynitrite. *SOD* neutralizes O_2_^−^ by converting it to H_2_O_2_, thus preventing its reaction with NO and increasing the half-life and bioavailability of NO [64,78].

The generation of excess ROS is thought to be of one of the primary mechanisms by which ionizing radiation causes tissue damage. Dr. M. Delp [79] and others propose a model wherein microgravity and radiation promote the generation of ROS in the resistance vasculature of bone that creates an imbalance favoring peroxynitrite over NO production. This lowers the bioavailability of NO, diminishes endothelium-dependent vasodilation, and disrupts the coupling of bone circulation with bone remodeling. While more studies are required to test the validity of this model, results from our group and others, as described in the succeeding sections, appear to be consistent with such a model.

### 3.2. Bone and Vascular Function during Development Are Intimately Associated

Vasculature is critical for normal skeletal development, postnatal growth, remodeling, and fracture repair. The embryonic skeleton undergoes endochondral ossification (reviewed in [80,81]) where cartilage, an avascular tissue, is gradually converted into bone, one of the most vascularized tissues in vertebrates [80]. This process occurs through a series of complex and coordinated signals that induce terminal differentiation of chondrocytes and their subsequent death. The extracellular matrix produced by the chondrocytes is then mineralized and partly degraded by chondroclasts and preosteoclasts, thus promoting the invasion of blood vessels. Following vascular invasion, osteogenic progenitors are recruited to the site, where they form mineralized bone [81]. A similar mechanism occurs during postnatal growth of the long bones, fracture repair and bone remodeling, although smaller in scale than what occurs during development. There is evidence that re-vascularization in response to bone injury is impaired in spaceflight. Rats that underwent osteotomy and then exposed to microgravity display reduced angiogenesis at the site of injury [82]. Since proper repair of skeletal tissue requires competent vascular invasion, this raises concerns of a higher risk for impaired fracture healing in individuals as a consequence of spaceflight.

The coupling of vascular perfusion and skeletal function has been demonstrated in a number of studies [83,84,85]. In addition, administration of anti-osteoporotic bisphosphonates improved blood flow and angiogenesis in aged rodents [86] while endochondral bone formation and angiogenesis are impaired in mice lacking certain isoforms of the pro-angiogenic signal, vascular endothelial growth factor (*VEGF*) [87]. Increased *VEGF* levels from over-expression of hypoxia-inducible factor alpha (*HIFα*) promotes both angiogenesis and osteogenesis, while loss of *HIF1a* in osteoblasts resulted in thinner and less vascularized bones [88].

### 3.3. Vascular-Bone Coupling Occurs via Redox-Dependent Mechanisms: Implications for Tissue Responses to Spaceflight

Cardiovascular deconditioning is one of the potential health risks associated with spaceflight [89,90]. A majority of astronauts experience orthostatic intolerance upon return from long-duration flight (129–190 days) [89,91,92,93] which is attributed to impairments in raising peripheral resistance [79]. Muscle vasculature plays an integral role in elevating peripheral resistance and, therefore, has been the subject of a number of studies using spaceflight models. In rodents, both spaceflight and hindlimb unloading impair vasoconstriction of gastrocnemius resistance arteries [94,95]. In addition, resistance arteries of mouse gastrocnemius [96] and rat soleus [97] both display defects in endothelium-dependent vasodilation. In rats, the soleus resistance arteries exhibit diminished expression levels of *eNOS* and *SOD1*, although the gastrocnemius resistance arteries do not show such alterations [97,98]. The basis for the site-specific differences in redox status of vascular resistance arteries is not fully understood. However, there is speculation that this is partly due to region-specific differences in perfusion and/or differences in metabolic rates of slow versus fast twitch muscle fibers and the relative abundance of these fiber types in the two muscle sites.

Similar to the observations in muscle resistance arteries, the vasculature of bone also exhibits functional impairments under spaceflight conditions. Two weeks of HU in rats results in deficits in vasoconstrictor and vasodilator properties of the femoral principal nutrient artery (PNA) [99], the primary route for blood circulation to the femur. HU animals display decrements in bone and marrow perfusion and increases in vascular resistance. These changes are not attributed to enhanced vasoconstrictor responsiveness of the bone resistance arteries, but are associated with decreased endothelium-dependent vasodilation and vascular remodeling that reduces PNA maximal diameter.

The skeleton, like muscle, undergoes deconditioning during spaceflight (and its analogs) which manifests as a reduction in bone mass due to a transient net increase in resorption [100,101,102] as well as diminished mechanical strength [4]. These coincide with functional impairments and changes in redox status in associated vasculature [93]. Greater decrements in percent cancellous bone volume were observed after 13 to 16 days of HU and exposure to simulated space radiation when treatments were combined, compared to untreated controls. HU reduced trabecular thickness, whereas irradiation reduced trabecular number but not thickness, accounting for the sometimes greater deficit in percent cancellous bone volume when HU and radiation are combined.

In the gastrocnemius feed artery, which served as a surrogate artery for the PNA, the early effects of HU and IR (^56^Fe) are to each impair peak endothelium-dependent vasodilation, with the combination of HU and IR exacerbating this deficit. These group differences are abolished in the presence of nitric oxide synthase (*NOS*) inhibitors, indicating that the impairment induced by HU and IR was mediated through the *NOS* signaling pathway. Vasodilation response to the NO donor DEA-NONOate is also impaired by HU and IR, indicating that the depressed endothelium-dependent vasodilation could be mediated in part through a reduced smooth muscle responsiveness to NO. Further, peak vasodilation correlates positively with percent cancellous bone volume which suggests a coupling of bone and vasculature responses to stressors associated with spaceflight. In contrast to vasodilator responses, HU and IR as single treatments or combined have little effect on vasoconstrictor properties or pressure-diameter responses. HU and HU with IR results in decreased levels of *eNOS* protein, while IR and HU with IR leads to diminished superoxide dismutase-1 (*SOD1*) and higher xanthine oxidase (*XO*) protein content. Decrements in the bioavailability of NO via reduction in *eNOS* protein levels, lower anti-oxidant capacity (*SOD1*) and higher pro-oxidant capacity (*XO*) may contribute to the deficits in *NOS* signaling in resistance arteries.

When the vascular effects of long-term (6–7 months) recovery from HU, IR (^56^Fe), and the combination of HU and IR are examined, only IR sustains an impairment in peak endothelium-dependent vasodilation [103]. The IR-induced deficit in endothelial vasodilator function is abolished by chemical inhibition of *NOS*, indicating that the sustained dysfunction is mediated through the *NOS* signaling pathway. Similar to the findings from an early timepoint (two weeks of HU with or without IR) [93], HU and IR have little effect on vasoconstrictor properties. These findings indicate that although both simulated weightlessness and irradiation produce early effects of impaired vascular endothelial function, only those produced by IR are sustained.

IR and HU causes differential effects on loss and thinning of trabeculae, contributing to lower percent cancellous bone volume when the treatments were combined. Both treatments impair endothelium-dependent vasodilation of skeletal muscle resistance arteries via a NO-dependent signaling pathway. The impairment of *NOS* signaling, however, seems to be differentially affected by unloading and irradiation; HU diminishes vascular *eNOS* protein expression whereas IR reduces *SOD* expression and increases pro-oxidant *XO* protein levels. If these findings in skeletal muscle resistance arteries hold for bone vasculature, impairments in endothelium-dependent vasodilation may lead to reductions in skeletal perfusion and perturbations in the coupling of bone cell and vascular endothelium activity.

### 3.4. Cellular Defenses to Oxidative Damage Are Important for Preserving Skeletal and Vascular Health

Most aerobic organisms have multiple defenses against the damaging effects of ROS, which include enzymatic and non-enzymatic antioxidants. Studies on perturbation of key antioxidant molecules and signaling pathways highlight the importance of antioxidant defenses in preserving the functional integrity of tissues and cells. In the following section, we summarize the state of knowledge on the role of endogenous antioxidant proteins in maintaining skeletal and vascular health with an emphasis on studies using genetic models of gain or loss of protein function. The cytoprotective effect of these proteins on Earth has, therefore, formed the rationale for investigating their importance in tissue and cellular defenses against the stressors associated with spaceflight [18,24] and its Earth-based analogs [34,35]. In this section, we also cite studies that illustrate the emerging role of these antioxidant proteins in modulating skeletal and vascular responses to spaceflight.

#### 3.4.1. Nuclear Factor Erythroid 2-Related Factor 2 (*NRF2*)

*NRF2* is a master transcription factor that regulates cellular redox balance and protective antioxidant and detoxification responses across multiple species, from *Drosophila* to humans [104,105,106]. It is a member of the Cap-N-Collar family of regulatory proteins composed of *NRF1*, *NRF3* and *BACH1* and *BACH2* [106] and is ubiquitously expressed with the highest concentrations occuring in heart, muscle and brain [107]. Under normal conditions, *NRF2* is sequestered to the cytosol via binding to its inhibitor, *KEAP1*, and is therefore subject to proteosomal degradation. During conditions of excess ROS, *NRF2* dissociates from *KEAP1* and undergoes translocation into the nucleus. *NRF2* then binds to antioxidant response-element sequences in the genome to promote transcription of hundreds of antioxidant genes [108] including *SOD1* as well as phase II detoxification enzymes [106].

In *Drosophila*, loss-of-function mutations in *dKEAP1*, an endogenous inhibitor of the *NRF2* fly homolog *CncC*, extends lifespan and increases resistance to the oxidizer paraquat [104]. Mice in which *NRF2* is globally deleted are viable, fertile and exhibit no apparent phenotypic defects [109,110]. However, consistent with its cytoprotective function, *NRF2* deletion in mice leads to increased sensitivity to radiation, oxidative damage and age-related disease pathologies [105,111]. Specifically, *NRF2* knockouts display accelerated heart failure in a myocardial infarct model [112], enhanced retinal degeneration [113,114], bone loss [110,115,116], and deficits in bone and endothelial stem and progenitor cell populations [115,117].

A number of studies have characterized the skeletal phenotype of *NRF2* knockouts and show that the effects of *NRF2* appear to be sex- and age-dependent. Cultured osteoclasts from *NRF2* knockout mice display elevated ROS levels, increased maturation and defective production of antioxidant enzymes. On the other hand, pre-treatment with *NRF2* agonists sulforaphane and curcumin, inhibit osteoclast maturation via activation of *NRF2* [118]. In female rodents, *NRF2* deletion results in decrements in percent cancellous bone volume and trabecular number as well as increased trabecular separation, which persists up to eight months of age but are no longer apparent thereafter. In addition, primary cultures of bone marrow-derived stromal cells from these KOs show diminished colony formation with no differences in osteoblast proliferation in vivo. Similarly, ex vivo osteoclast cultures from *NRF2* KOs mature faster than those from wild-type animals, although in vivo osteoclast counts are unchanged [115]. In contrast, *NRF2* deletion in male rodents leads to higher bone mass, mineral apposition rate and osteoblast number compared to wild-type controls [119]. These sex-dependent differences were corroborated in a more rigorous study comparing male and female *NRF2* KOs [110]. *NRF2* deficiency in females results in reduced femoral and spinal bone mineral density, while loss of *NRF2* in males leads to an improvement in the said bone structural parameters compared to sex-matched controls. Furthermore, both young (3-month old) and old (15-month old) KO females display decreased expression of *NRF2* target antioxidant enzymes; in contrast, these deficits are only observed in aging males [110].

Collectively, the abovementioned studies demonstrate that *NRF2* plays a critical role in maintaining the functional and structural integrity of bone and vasculature here on Earth, and that loss of the *NRF2* gene mimics some of the features of spaceflight-induced bone loss. However, how *NRF2* impacts tissue responses to elements of the space environment (radiation or microgravity) or its analogs is less clear. One study determined the consequence of *NRF2* loss in a rodent radiotherapy model at high doses (20 Gy) of ionizing radiation [116] where it was found that *NRF2* ablation exacerbates bone loss and impairs colony-forming capacity of bone marrow-derived progenitors [116]. Likewise, the role of *NRF2* in tissue responses to microgravity has not been thoroughly examined, although there is some evidence of its function in mechanical load-driven bone formation. *NRF2* KOs are less responsive to the anabolic effects of mechanical loading of the ulna compared to wild-type controls, as indicated by blunted bone formation rate and decreased relative mineralizing surface. In addition, cultured primary osteoblasts from mechanically loaded ulna of *NRF2* KOs display reductions in the expression levels of antioxidant enzymes [120].

Microgravity causes a cephalad fluid shift [121] as well as changes in ion homeostasis [122,123,124] and there is indirect evidence that *NRF2* may play a role in the endothelial response to perturbations in ionic balance. *NRF2* KO and wild-type controls were fed either a low or high salt diet and vascular function was analyzed. Endothelium-dependent dilation to acetylcholine is unchanged in the middle cerebral arteries (MCA) in both groups fed a low-salt diet. High-salt diet eliminates endothelium-dependent dilation to acetylcholine in both genotypes. However, unlike wild-type controls, *NRF2* KO rats fail to respond to the rescuing effects of angiotensin II infusion on high-salt diet-induced endothelial dysfunction and microvessel rarefication [125].

#### 3.4.2. CuZn Superoxide Dismutase (*SOD1*)

*SOD1* gene expression is downregulated in hair follicles of spacefight crew members [24] while its protein levels are decreased in livers of spaceflown rats [18] although its role in protecting vasculature and bone from spaceflight stressors needs to be further elucidated. *SOD*s are the major antioxidant defense systems against O_2_^−^, catalyzing the conversion of O_2_^−^ to H_2_O_2_, which is then further reduced to water by catalase, peroxiredoxins (PRx), or glutathione peroxidases (*GPX*) [64]. In mammals, the *SOD* family is comprised of three isoforms: cytoplasmic CuZnSOD (*SOD1*), mitochondrial MnSOD (*SOD2*), and the extracellular CuZnSOD (*SOD3*), and as their names indicate, require a metal co-factor (Cu, Mn, or Zn) for their activation. Both male and female *SOD1* knockout mice exhibit increased oxidative stress, and decreased bone mineral density and mechanical strength (bending stiffness) as assessed by three-point bending compared to sex-matched wild type controls [126]. Consistent with the observations in bone, *SOD1* deletion also leads to increased oxidative damage in skeletal muscle, accelerates age-dependent skeletal muscle atrophy and motor function deficits as assessed by voluntary wheel running and rotating rod test [127]. Similar to results from deletion of its transcriptional regulator *NRF2*, loss of the *SOD1* gene leads to musculoskeletal deficits, also resembling some of tissue deficits observed in spaceflight and its Earth-based analogs.

#### 3.4.3. Catalase

Catalase is an anti-oxidant that converts the reactive species, H_2_O_2_, into water and molecular oxygen. Transgenic mice over-expressing the human catalase (*hCAT*) gene targeted to the mitochondria (*mCAT* mice) display extended lifespan [128] and reductions in numerous pathologies [129,130,131] including cardiovascular disease [131,132,133] and Alzheimer-related amyloid deposition [130]. Further, the transgene effectively quenches mitochondrial oxidative stress in macrophages which are precursors to bone-resorbing osteoclasts [134]. Consistent with the latter finding, over-expression of catalase targeted to mitochondria of bone-resorbing osteoclasts protects from bone loss caused by the loss of estrogens [135]. In addition, the transgene rescues cardiomyopathy including impaired systolic and diastolic function in mice that carry homozygous mutations in the exonuclease encoding domain of mitochondrial DNA polymerase γ (Polg(m/m)) [131].

Catalase protein levels and activity are decreased in livers of spaceflown rats [18]. Furthermore, a number of studies involving the mitochondrial *hCAT* transgene demonstrate the importance of mitochondrial ROS quenching in the cellular defense against HZE particles. *mCAT* animals display protection from proton-induced deficits in synaptic signaling, dendritic complexity, memory and cognition [136,137,138]. The impact of mitochondrial ROS quenching on skeletal and cardiovascular responses to microgravity and radiation is less understood and is currently under investigation by our team and others.

## 4. Implications for the Development of Spaceflight Countermeasures

Whole body irradiation increases ROS generation by bone marrow cells [33,139], damages skeletal lipids [33], and upregulates the activity of the antioxidant enzyme *NRF2* in both bone marrow and mineralized tissue [35,140]. On the other hand, *NRF2* deficiency can exacerbate radiation-induced bone loss [116]. Collectively, these data suggest that pharmacologic interventions directed toward limiting excess ROS may moderate the radiation stress response of bone cells.

Within a period of three days to a month, relatively low doses of radiation (≤2 Gy) lead to progressive loss of cancellous bone, as shown by our group and others [34,35,141,142,143,144], which is preceeded by a rapid increase in expression levels of inflammatory cytokines such as *IL1* and *TNFα* and pro-osteoclastogenic signals *RANKL* and *MCP1* as well as the oxidative defense gene, *NRF2* [34,35]. On the basis of these findings, we hypothesized that diets or drugs capable of preventing early increases in these molecular signals can mitigate cancellous bone loss caused by both low LET and high LET radiation. The hypothesis was tested by evaluating a number of candidate interventions including: (1) an antioxidant diet cocktail (AOX) composed of *N*-acetyl cysteine, ascorbic acid, l-selenomethionine, dihydrolipoic acid and vitamin E which in combination has been shown to protect a variety of tissues from ionizing radiation [145,146,147]; (2) dihydrolipoic acid (DHLA), which possesses antioxidant properties [148,149]; (3) the non-steroidal anti-inflammatory drug, Ibuprofen [150,151]; and (4) Dried Plum (DP, 25% by weight), previously reported to inhibit osteoclast activity and protect from age-related bone loss [152,153,154,155]. Findings from this study indicate that high levels of pro-resorptive, pro-inflammatory, and oxidative stress-related genes in bone marrow strongly correlate with cancellous bone loss. DP diet completely prevents radiation-induced increases in these molecular signals and the ensuing cancellous bone loss [35] which appears to strengthen the rationale for using antioxidants to mitigate radiation-induced bone loss. However, seemingly paradoxical is that the other two antioxidant-based interventions, DHLA and AOX, failed to protect from bone loss induced by radiation. These findings do not nullify the feasibility of using antioxidants as a countermeasure, yet highlight very important considerations in countermeasure design for radiation-induced bone loss. Firstly, an effective strategy to mitigate radiation-induced bone loss must at least include components that prevent the early rise in expression of pro-resorptive, pro-inflammatory, and oxidative stress-related genes. This knowledge is gained from the observation that treatments which fail to mitigate the changes in all these three molecular signals (such as AOX and Ibuprofen) ultimately were unsuccessful in preventing radiation-induced bone loss. Secondly, there are additional, equally important signaling molecules likely to mediate radiation-induced bone loss. This is a lesson learned from the finding that DHLA, which appeared nearly as effective as DP in preventing radiation-induced increases in expression of these markers, did not protect skeletal microarchitecture. Lastly, an assessment of the efficacy of an intervention on multiple organ systems must be incorporated in countermeasure development efforts as antioxidants appear to confer various levels of protection in different tissues.

## 5. Concluding Statements

**Figure 1:**
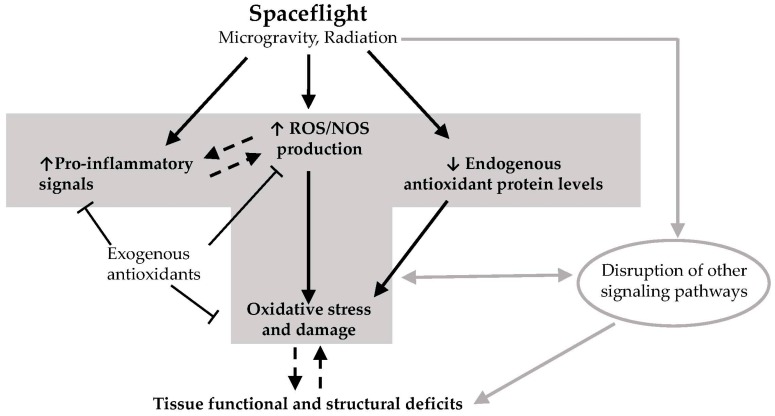
Hypothetical model on how spaceflight leads to deficits in tissue function and structural integrity. Exposure of tissues to elements of the spaceflight environment such as microgravity and radiation (and potentially other unknown factors) leads to enhanced production of reactive oxygen species (ROS) and reactive nitrogen species (NOS), increased levels of pro-inflammatory signals, and downregulation of endogenous antioxidant defenses. This leads to excess ROS/NOS due to an imbalance between endogenous antioxidant protein levels and ROS/NOS production. Excess ROS/NOS leads to oxidative damage of proteins, lipids and DNA which in turn result in deficits in tissue function and structural integrity. Other non-redox signaling processes may also contribute to these deficits. Some exogenous antioxidants found in the diet may block the increases in ROS/NOS levels and inflammatory signals, thereby preventing oxidative damage. It remains to be elucidated whether inflammation causes ROS production and/or vice versa in the context of spaceflight, and whether oxidative damage mediates or results from progressive tissue degeneration as a consequence of spaceflight. T-bar arrow: inhibitory effect; dotted line arrow: cause and effect needs further elucidation. Gray arrows depict the contribution of non-redox related processes in spaceflight-induced deficits in tissue structure and function.

Studies overwhelmingly demonstrate a link between oxidative damage and tissue dysfunction that ensues from exposure to spaceflight factors (Figure 1). One of the outstanding questions that needs to be addressed is whether oxidative damage mediates or results from progressive tissue degeneration in the course of spaceflight [156]. More studies using genetic models for altered redox status will contribute to resolving this question. Future interplanetary travel will increase the duration of exposure of humans to spaceflight. Therefore, additional comprehensive investigations involving extended timepoints that simulate the duration of long-term missions and the ensuing recovery must be conducted to better estimate health risks to crew. Furthermore, the latent effects of spaceflight on the cardiovascular system and on stem and progenitor cells of the bone marrow must be examined for their potential to exacerbate the effects of aging. The use of antioxidant-based countermeasures must be guided by the understanding that low levels of ROS signaling are important for normal cellular function. Therefore, future studies must also assess the potential of any antioxidant-based countermeasure to perturb these essential signaling events and their consequences on the functional integrity of tissues. Research progress on the aformentioned areas will help in the development of effective approaches for maintaining crew health during and after interplanetary missions.
